# Marco Breschi, Lorenzo Del Panta (eds.), Carlo Corsini: saggi di vita. [Carlo Corsini:life essays], Forum:Udine, 2018, ISBN: 978-88-3283-109-2

**DOI:** 10.1186/s41118-018-0050-x

**Published:** 2019-01-07

**Authors:** Alessio Fornasin

**Affiliations:** 1 University of Udine, Udine, Italy; 2 University of Trieste, Trieste, Italy

The book, published in memory of Carlo Corsini (1935–2017), Professor Emeritus of Demography at the University of Florence, contains a selection of his works published over more than 40 years of activity. The studies well represent his multiform and original personality as a researcher and retrace some of the most significant stages of his scientific career. The book collects ten papers and covers all his main areas of research. It illustrates the wide range of the author’s interests, highlights his strong inclination towards interdisciplinarity and demonstrates his ability to enhance the sources, not only with the skilful use of the technical tools of the demographer and the historian, but, above all, with an inexhaustible number of research questions.

After the editors’ introduction, which traces Corsini’s scientific career and life history, the collection opens with an article on infant mortality, published in 1966 on this journal, that it may be considered his first paper in historical demography. The paper compares mortality in the first year of life in three Italian regions: Liguria, Piedmont and Tuscany, during the Napoleonic period.

The subsequent paper is focused on the same historical period too. It is a paper on seasonal migration in the Italian departments of the French Empire, published in 1969. In this broad essay, he makes use of documents of a Napoleonic survey on the workers’ mobility. These migrations involved tens of thousands of men who each year left their homes to work away from their families and communities. Corsini masterfully deals with the subject and studies migration, putting it in constant relation with other demographic factors.

Another strand of Corsini’s studies represented in the book is the local communities’ demographic history. In *Ricerche di demografia storica del territorio di Firenze* (*Historical demography research on the territory of Florence*, 1975), he investigates some aspects of marriage and fertility of three communities in the Florentine countryside (Fiesole, San Godenzo and Empoli) through the family reconstitution technique. Even the essay *Le trasformazioni demografiche e l’assetto sociale* (*Demographic change and social structure*, 1988) about the city of Prato belongs to this research topic. The period covered by the paper goes from the end of the Napoleonic era to the Second World War. The study is, among Corsini’s works, perhaps the one not only in which he develops with greater consistency the “classic” historical demography analysis, but where demographic aspects are merged into a unitary framework with the social characteristics of the community in its historical context. In this comprehensive study, it is a monograph for dimension, Carlo Corsini not only demonstrates his skill in describing, in an original and very lively way, the development of the various components of the city’s demographic evolution (fertility, nuptiality mortality), but also deals with more particular aspects, such as the abandoned childhood. Finally (perhaps the most stimulating part of the essay), he studies the evolution of the socio-economic structures of Prato, with particular attention to social mobility, analysed on the basis of a sample of people followed through various censuses.

Two other essays give an account of the interest that Carlo Corsini has always had on the theme of marriage. In the first, *Uomini saggi, femmine folli* (*Wise men, mad women*, 1980), he focused on second weddings. The study deals with the theme of the marriage market in eighteenth-century Tuscany with continuous references to popular proverbs. The work is written in a balance between demographic analysis and cultural history and shows Corsini’s aptitude to broaden the boundaries of demography to include other themes and other reflections. At the end of the paper, we find a sort of manifesto of his scientific thought: “Every demographic phenomenon – better said: human – has explanations that can be either purely demographic, or social or, more generally, ‘cultural’. Moreover, even phenomena that simply appear to be of a cultural nature raise demographic problems”.

The paper *Chi si sposa per primo?* (*Who gets married first?*, 2003), instead, analyses and interprets in different historical and social contexts the possible link between order of birth and order of marriage. The paper contains numerous and stimulating literary references (from Shakespeare to the Bible, from Jane Austen to Italo Calvino) about rules, customs and traditions that often conditioned, within a family, the access to marriage of sons and daughters.

This book could not miss some essays dedicated to the subject that, perhaps, more than any other has permeated Corsini’s activity: abandoned childhood. Two papers, respectively, of 1991 and 1998, well represent the mastery with which he has always treated the various facets of this topic. The papers are based mainly on the documentary collections of the *Spedale degli Innocenti* (the hospital for abandoned children), but as always, in Corsini, the information is crossed with other documentary collections, in order to observe the phenomenon in its more general context. In a third paper on the subject, *Materiali per lo studio della famiglia in Toscana* (*Materials for the study of the family in Tuscany*, 1976), he addresses the issue from the perspective of abandoned children who are also legitimate children. The paper, from a methodological point of view, anticipates those researches which, relying on large datasets, link many information from different sources collected at an individual level.

The book closes with an essay in which Carlo Corsini reflects on the role of the family in society (2009). This is an essay that well represents the synthesis of his thought and his interests. In fact, he has always tried to focus on the family from various perspectives and in different contexts, treating with equal interest and attention both the aspects of the method and the problems of field research, always directing it towards the margins and the most hidden interstices of society.

Finally, it can be said that the book is not just a celebration of a great scholar. Some themes, in fact, are very topical and the collected papers still represent important studies for the understanding of phenomena such as infant mortality, marriage choice, emigration.... The book is not only interesting for scholars of historical demography, but also, and above all, it is suitable for all demographers, especially the younger ones. This is true not only for the issues covered, but for the author’s approach, certainly very personal, to scientific research, made of curiosity, attention to detail and proliferation of new strands of investigation, which is one of the most important legacies of Carlo Corsini, demographer and historian.

